# Friedreich's Ataxia Causes Redistribution of Iron, Copper, and Zinc in the Dentate Nucleus

**DOI:** 10.1007/s12311-012-0383-5

**Published:** 2012-05-05

**Authors:** Arnulf H. Koeppen, R. Liane Ramirez, Devin Yu, Sarah E. Collins, Jiang Qian, Patrick J. Parsons, Karl X. Yang, Zewu Chen, Joseph E. Mazurkiewicz, Paul J. Feustel

**Affiliations:** 1 Research Service (151), Veterans Affairs Medical Center, 113 Holland Ave, Albany, NY 12208 USA; 2 Department of Neurology, Albany Medical College, Albany, NY 12208 USA; 3 Department of Pathology, Albany Medical College, Albany, NY 12208 USA; 4 Laboratory of Inorganic and Nuclear Chemistry, Wadsworth Center, New York State Department of Health and Department of Environmental Health Sciences, Albany, NY 12201 USA; 5 The University at Albany, Albany, NY 12201 USA; 6 X-Ray Optical Systems, East Greenbush, NY 12061 USA; 7 Center for Neuropharmacology and Neuroscience, Albany Medical College, Albany, NY 12208 USA

**Keywords:** Cu^++^-transporting ATPase α-peptide (ATP7A, Menkes protein), Copper, Cu,Zn-superoxide dismutase, Dentate nucleus, Ferritin, Friedreich's ataxia, Iron, Metallothionein, X-ray fluorescence, Zinc

## Abstract

Friedreich's ataxia (FRDA) causes selective atrophy of the large neurons of the dentate nucleus (DN). High iron (Fe) concentration and failure to clear the metal from the affected brain tissue are potential risk factors in the progression of the lesion. The DN also contains relatively high amounts of copper (Cu) and zinc (Zn), but the importance of these metals in FRDA has not been established. This report describes nondestructive quantitative X-ray fluorescence (XRF) and "mapping" of Fe, Cu, and Zn in polyethylene glycol–dimethylsulfoxide (PEG/DMSO)-embedded DN of 10 FRDA patients and 13 controls. Fe fluorescence arose predominantly from the hilar white matter, whereas Cu and Zn were present at peak levels in DN gray matter. Despite collapse of the DN in FRDA, the location of the peak Fe signal did not change. In contrast, the Cu and Zn regions broadened and overlapped extensively with the Fe-rich region. Maximal metal concentrations did not differ from normal (in micrograms per milliliter of solid PEG/DMSO as means ± S.D.): Fe normal, 364 ± 117, FRDA, 344 ± 159; Cu normal, 33 ± 13, FRDA, 33 ± 18; and Zn normal, 32 ± 16, FRDA, 33 ± 19. Tissues were recovered from PEG/DMSO and transferred into paraffin for matching with immunohistochemistry of neuron-specific enolase (NSE), glutamic acid decarboxylase (GAD), and ferritin. NSE and GAD reaction products confirmed neuronal atrophy and grumose degeneration that coincided with abnormally diffuse Cu and Zn zones. Ferritin immunohistochemistry matched Fe XRF maps, revealing the most abundant reaction product in oligodendroglia of the DN hilus. In FRDA, these cells were smaller and more numerous than normal. In the atrophic DN gray matter of FRDA, anti-ferritin labeled mostly hypertrophic microglia. Immunohistochemistry and immunofluorescence of the Cu-responsive proteins Cu,Zn-superoxide dismutase and Cu^++^-transporting ATPase α-peptide did not detect specific responses to Cu redistribution in FRDA. In contrast, metallothionein (MT)-positive processes were more abundant than normal and contributed to the gliosis of the DN. The isoforms of MT, MT-1/2, and brain-specific MT-3 displayed only limited co-localization with glial fibrillary acidic protein. The results suggest that MT can provide effective protection against endogenous Cu and Zn toxicity in FRDA, similar to the neuroprotective sequestration of Fe in holoferritin.

## Introduction

In the vast majority of patients with Friedreich's ataxia (FRDA), the mutation consists of a homozygous guanine–adenine–adenine (GAA) trinucleotide repeat expansion in the frataxin gene (chromosome 9q21). The normal gene product, frataxin, is critically important for the biogenesis of iron–sulfur clusters though the protein may have several other functions (review in ref. [1]). Among other lesions of the central and peripheral nervous systems, FRDA causes selective atrophy of the large neurons of the dentate nucleus (DN) [2]. Smaller nerve cells persist [2] and continue to provide GABA-ergic afferent terminals to the inferior olivary nuclei. In contrast, GABA-ergic corticonuclear connections that normally provide two thirds of synaptic terminals to the DN, undergo grumose degeneration, and the overall synaptic density declines [3]. A recent systematic anatomical study established that loss of large DN neurons constitutes the only critical interruption of the "cerebellar module" in FRDA [2] and the main correlate to cerebellar ataxia in FRDA.

The DN normally contains abundant iron (Fe). Peculiarly, collapse of this gray matter structure in FRDA does not lead to a net decrease or increase of the metal when concentrations are determined on tissue digests [4]. The vulnerability of the DN to frataxin deficiency may or may not be related to its high Fe content. Several other gray matter structures, such as globus pallidus, subthalamic nucleus, red nucleus, and substantia nigra, are also Fe-rich but appear exempt from destruction in FRDA. To date, there is no direct evidence that Fe in the DN becomes toxic or that the lesion is due to an excess of reactive oxygen species. Nevertheless, many in vitro models of FRDA show great sensitivity to oxidative stress. Bayot et al. [5] recently summarized the evidence for the primary role of Fe in the pathogenesis of FRDA and proposed that Fe accumulation in certain systems, including the human heart, is a secondary event. Though Fe may not be critical in the damage to large DN neurons, the metal may be important in downstream effects of the primary lesion. Koeppen et al. [4] documented changes in two Fe-responsive proteins of the DN, ferritin and ferroportin, by immunohistochemistry and analysis of ferritin subunits by Western blotting. The results suggested that Fe does not remain inert throughout the course of FRDA [4], implying the presence of ionic iron as a potential catalyst in the Fenton reaction.

The DN also contains two other metals with established physiological functions, namely, copper (Cu) and zinc (Zn). The concentrations of these metals are relatively high at 10–15 % of total Fe [6–9]. A prior synchrotron study of fixed unembedded slices of human cerebellum confirmed prominent fluorescence of Fe, Cu, and Zn in the DN but also showed that these metals are only partially co-localized [10]. In contrast to Fe, the literature contains no information heretofore on the fate of Cu and Zn in the DN of patients with FRDA. Inappropriate release of endogenous Cu ions, and perhaps Zn ions, from DN neurons may contribute to oxidative injury. The combined effect of free Fe and Cu is perhaps more serious than that of either metal alone. The experimental intracerebral, intraventricular, or subarachnoid injection of inorganic Cu salts [11, 12] is very destructive, but no data are available regarding the cells that regulate the metal or the response of specific cuproproteins. The experimental intraventricular administration of an *organic* Cu compound, namely, Cu-histidine, in an effort to explore potential replacement therapy in Menkes disease, also causes damage to the exposed brain surfaces [13].

This report presents qualitative and quantitative observations on the DN in FRDA that were gained through application of nondestructive X-radiation of tissue samples. X-ray fluorescence (XRF) of Fe, Cu, and Zn was correlated with the histopathology of the DN. While ferritin is an excellent marker of Fe dysmetabolism, similar storage proteins for Cu and Zn do not exist. This effort also included the immunohistochemical examination of two cuproproteins that may "recognize" shifts in brain Cu levels, namely, Cu,Zn-superoxide dismutase (SOD) and Cu^++^-transporting ATPase α-peptide (ATP7A, Menkes protein), and of three metallothionein isoforms.

## Material and Methods

### Tissue Samples and Embedding in Polyethylene Glycol 1450/Dimethylsulfoxide

Table 1 shows basic clinical and genetic information of 10 patients with FRDA from whom sufficient DN tissue was available for embedding in polyethylene glycol 1450/dimethylsulfoxide (PEG/DMSO) and examination by XRF. Control samples came from 13 persons (3 women, 10 men) who died without evidence of central nervous system disease. Mean age of death in years and standard deviation were 68.7 ± 10.5 (range 50–85 years). The authors received approval from the Institutional Review Board at the Veterans Affairs Medical Center in Albany, NY, USA, for research on autopsy tissues obtained from human subjects.

**Table 1:** Basic clinical information of 10 patients with FRDA

Patient no. and sex	Age of onset (years)	Age of death (years)	Disease duration (years)	GAA repeats allele 1	GAA repeats allele 2
FRDA 1, M	10	24	14	1,050	700
FRDA 2, M	7	34	27	1,114	1,114
FRDA 3, F	17	50	34	1,122	515
FRDA 4, F	50	83	33	236	106
FRDA 5, F	18	63	45	730	639
FRDA 6, F	6	23	17	864	668
FRDA 7, M	9	33	24	925	925
FRDA 8, M	9	27	18	1,070	700
FRDA 9, F	15	69	54	560	560
FRDA 10, M	25	67	42	160	160
	16.6 ± 13.1^a^ (6–50)	47.3 ± 21.9 (24–83)	30.8 ± 13.2 (14–54)	783 ± 357 (160–1,122)	609 ± 306 (106–1,114)

^a^Mean ± standard deviation and range

Fixed pieces of cerebellar hemisphere with distinctly visible DN were progressively infiltrated by PEG (Sigma, St. Louis, MO, USA) as described before [14, 15]. Water and fixative in the tissue samples were replaced at room temperature by immersion in PEG 400 at increasingly higher concentrations (30–90 % by volume), followed by PEG 1000 and PEG 1450/DMSO at 60°C. After cooling, the tissue blocks were "faced" in a microtome to present a smooth surface for the measurements of Fe, Cu, and Zn by XRF. Care was taken to prevent metal contamination by use of Teflon-coated microtome blades (Sturkey, Lebanon, PA, USA).

### Monochromatic XRF Instrumentation

**Fig. 1:**
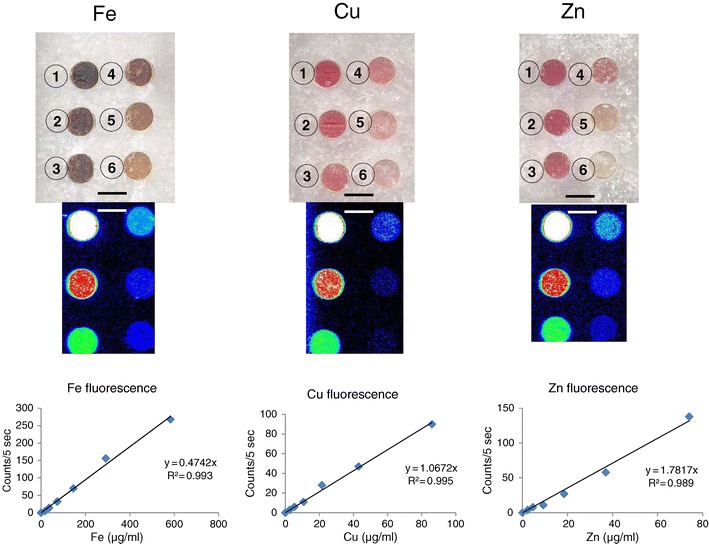
Fe, Cu, and Zn standards. *Top panel*: Congealed solutions of Fe-III-, Cu-II-, and Zn-II-mesoporphyrins in gelatin capsules. The highest concentrations are present in ① of each standard. After dilution, the concentrations range, in micrograms per milliliter, from 620 to 19.375 for Fe; 86 to 2.688 for Cu; and 74 to 3.125 for Zn. *Middle panel*: Matching XRF maps of the standards shown in the top panel. Pseudocolors indicate the declining concentration from maximum (*white*) to red, green, light blue, and dark blue. *Bottom panel*: Regression analysis of Fe, Cu, and Zn. Ten point-measurements were made of each standard concentration and averaged. The mean values were reduced by background XRF that was obtained by sampling the block outside the capsules. The goodness of fit is given by *R* ^2^ values. *Bars* in top and *middle panels* represent 5 mm

A custom-built XRF mapping instrument was assembled by X-Ray Optical Systems (XOS, East Greenbush, NY, USA) specifically for use in this study and optimized for imaging Fe, Cu, and Zn distributions in PEG/DMSO-embedded tissue blocks. Chen et al. [16] and Gibson et al. [17] have published theory and practical application of nondestructive XRF. Hardware components include a 50-kV, 1-mA, molybdenum target X-ray source, generating a monochromatic beam through a doubly curved crystal optic (DCC) [18]; a motion controller with an *x*–*y* scanning mechanism; and a silicon drift detector (SDD). Specimens are mounted in a custom-designed holder that aligns the faced surface in a perfectly horizontal position. The instrument contains a small camera that allows the user to define the region on standards or specimens that are to be scanned. Multiple specimens can be scanned sequentially. The X-ray beam travels in a raster-like manner across the user-defined region of the samples, and Fe, Cu, and Zn fluorescent photons are detected and counted by the SDD. Step widths and exposure times can also be controlled by the operator. For this investigation, these parameters were set at 0.1 mm and 5 s, respectively. The source beam coupled with the DCC optic generates an elliptical spot of radiation that varies with the distance of the delivery optics from the surface of the specimen (the *z*-axis). In a typical application, it measures 100 × 240 μm (18,849 μm^2^ ≈ 0.02 mm^2^). The unit can be operated at room temperature without the need for a vacuum and has an overall power consumption of 350 W. The software application is a Windows-based computer program that generates two-dimensional images or "maps" of metal distribution, with X-ray intensity represented on the *z*-axis as a color-coded scale. XRF signals are recorded as counts/5 s, and signal intensity is directly proportional to metal concentration (Fig. 1).

### Calibration Strategy for Quantitative Measurements by XRF

Calibration standards for Fe, Cu, and Zn were prepared from pure compounds of each element, in which the metal ion is chelated by a porphyrin ring. Fe-III-, Cu-II-, and Zn-II-mesoporphyrins were purchased from Frontier Scientific, Logan, UT, USA. A known mass of each material was weighed out and the metal content estimated on the molecular formula of each compound, the relative atomic mass of each metal, and the known relative mass of the metalloporphyrin. The target concentrations of Fe, Cu, and Zn were estimated to be 600, 100, and 100 μg/ml, respectively. The metalloporphyrins were first dissolved in DMSO (Sigma, St. Louis, MO, USA). Hot liquid PEG 1450 (60°C) was then added to achieve a DMSO concentration of 10 %. A dilutional series was prepared by the addition of the liquid PEG/DMSO mixture, and each successive concentration of metal represented one half of the preceding mixture. Six equally spaced circular openings were made in a blank PEG/DMSO block with the aid of a 5-mm-wide cork borer (Fig. 1). The longer portions of no. 3 gelatin capsules (Star West Botanicals, Rancho Cordova, CA, USA; approximate volume, 450 μl) were then inserted to receive the hot solutions of serially diluted metalloporphyrins (about 400 μl; Fig. 1). The liquid transmitted sufficient heat to temporarily melt the surrounding solid PEG and provide a tight seal after cooling. After the PEG/DMSO mixtures became solid, capsules and surrounding block were faced as described above for tissue samples.

The exact concentrations of Fe, Cu, and Zn of the PEG-embedded XRF calibration standards were determined independently using another analytical technique. Briefly, separate gelatin capsules were prepared in which 100 μl of each of the metalloporphyrins in PEG/DMSO (the highest standard concentration) and unspiked PEG/DMSO (the calibration blank) were analyzed for Fe, Cu, and Zn by the Laboratory of Inorganic and Nuclear Chemistry at Wadsworth Center, New York State Department of Health. Each capsule was digested in concentrated nitric acid, hydrogen peroxide, and concentrated hydrochloric acid on a hot block using an established procedure. After appropriate dilution, Fe, Cu, and Zn were determined in the digest by inductively coupled plasma-optical emission spectrometry. The found values for Fe, Cu, and Zn in micrograms per milliliter of PEG/DMSO were 620, 86, and 74, respectively, and yielded six-point linear calibration curves (Fig. 1). Detection limits, in micrograms per milliliter (or parts per million), are 4 for Fe, 1 for Cu, and 1 for Zn.

Each run included the prepared Fe, Cu, and Zn standards and up to four tissue blocks containing DN from FRDA patients or controls. The regions of PEG/DMSO between the calibration standards (Fig. 1) and just outside the visible tissue samples served as blanks. Maps obtained from tissue samples were used to compare the localization of metals. In a typical run, signal strengths were divided into six intervals, ranging from background to maximum XRF count rate. This process generated six distinct "zones" of XRF intensity within the arbitrary interval limits that were indicated by six separate colors. In order of declining XRF strength, these colors were white, red, orange, green, light blue, and dark blue. Ten points in each zone were analyzed for Fe, Cu, and Zn content. The average count rate (counts/5 s) was corrected by subtracting the mean background signal, and the corrected XRF was used to quantify Fe, Cu, and Zn based on the respective calibration curves (Fig. 1).

### Tissue Recovery from PEG/DMSO

After XRF mapping and signal quantification were complete, the tissue blocks were immersed in phosphate-buffered saline (PBS), and PEG/DMSO was completely removed through several changes of PBS. The recovered tissues were then suspended overnight in sodium phosphate-buffered 4 % paraformaldehyde prior to embedding into paraffin by standard procedures.

### Immunohistochemistry, Immunofluorescence, and Lectin Affinity Fluorescence

Six micrometer-thick paraffin sections were stained by hematoxylin and eosin to exclude specimens with siderocalcinosis of the DN. Monoclonal or polyclonal antibodies to the following proteins were used for immunohistochemistry or immunofluorescence: neuron-specific enolase (NSE; mouse monoclonal; Chemicon, Temecula, CA, USA); glutamic acid decarboxylase (GAD; mouse monoclonal; MBL International, Woburn, MA, USA); ferritin (rabbit polyclonal; DAKO, Carpinteria, CA, USA; goat polyclonal, GenWay, San Diego, CA, USA); SOD (rabbit polyclonal; Sigma-Aldrich, St. Louis, MO, USA); ATP7A (rabbit polyclonal; Bioworld Technology, St. Louis Park, MN, USA); metallothioneins 1 and 2 (MT-1/2) (mouse monoclonal; Invitrogen, Frederick, MD, USA); metallothionein-3 (MT-3) (rabbit polyclonal; Sigma-Aldrich, St. Louis, MO, USA); glial fibrillary acidic protein (mouse monoclonal, Sternberger Monoclonals-Covance, Princeton, NJ, USA; and rabbit polyclonal, DAKO); and CD68 (monoclonal, Santa Cruz Biotechnology, Santa Cruz, CA, USA). The immunohistochemical protocol was similar to previously published techniques [2, 15]. Antigen retrieval methods were DIVA (a proprietary name for a decloaking solution sold by Biocare Medical, Concord, CA, USA) for NSE, GAD, glial fibrillary acidic protein (GFAP), ferritin, and the metallothioneins or incubation in 0.1 M citric acid/sodium citrate buffer (pH 6) for 15 min at 95°C for SOD, ATP7A, and CD68. Fluorophore-labeled secondary antibodies (Cy3 and Alexa488) were purchased from Jackson ImmunoResearch, West Grove, PA, USA. Biotinylated secondary antibodies and fluorescein isothiocyanate (FITC)-labeled streptavidin came from Vector Labs., Burlingame, CA, USA. Horseradish peroxidase-labeled streptavidin was purchased from Sigma-Aldrich, St. Louis, MO, USA.

Co-localization of SOD in ferritin-reactive cells of the DN was examined by double-label immunofluorescence with the goat polyclonal antibody to human ferritin and the rabbit polyclonal anti-SOD antibody. The technique was similar to a previously described method [4] though the blocking solution used here was 10 % normal donkey serum in PBS and antibodies were diluted in 1 % donkey serum. Chelation and antigen retrieval with DIVA were identical to the immunohistochemical protocol for the visualization of ferritin. After incubation with anti-ferritin, the affixed antibody was detected with Cy3-labeled donkey anti-goat IgG. After washing and additional suppression, SOD was visualized by the sequence: rabbit polyclonal anti-SOD → biotinylated anti-rabbit IgG → FITC-labeled streptavidin. Co-localization of SOD and CD68-positive microglia in the DN was determined by the following sequence: mouse monoclonal anti-CD68 → donkey Cy3-labeled anti-mouse IgG → rabbit anti-SOD → Alexa488-labeled anti-rabbit IgG. Images were analyzed in a Zeiss LSM 510 Meta confocal microscope with a PlanApo 63X oil immersion objective and a numerical aperture of 1.4. Exciting wavelengths alternated between 488 and 543 nm, and the images were collected through band pass (BP) filters of 500–530 nm for FITC and Alexa488 and 565–615 nm for Cy3.

The localization of ATP7A in vessel walls was based on lectin affinity cytochemistry with tetramethylrhodamine isothiocyanate (TRITC)-labeled *Ricinus communis* agglutinin 1 (RCA-1) (Vector) after immunofluorescence with polyclonal anti-ATP7A had been completed through the series of biotinylated anti-rabbit IgG and FITC–streptavidin. The labeled lectin was diluted in 0.05 M Tris buffer pH 7.6, containing 1 mM CaCl_2_, and applied at room temperature for 1 h. The sections were washed in the same CaCl_2_-containing buffer and then mounted with the same buffer containing 50 % glycerol (by volume). For visualization, the BP filters were set at 500–530 nm for FITC and 565–615 nm for TRITC.

Visualization of MT-1/2 and MT-3, and GFAP, by double-label immunofluorescence varied with the nature of the primary antibodies. For MT-1/2, detectable by a mouse monoclonal antibody, the sequence was (washing and blocking steps omitted): monoclonal anti-MT-1/2 → Alexa488-labeled donkey anti-mouse IgG → rabbit polyclonal anti-GFAP → Cy3-labeled donkey anti-rabbit IgG. For MT-3, detectable by a polyclonal antibody, the sequence was: monoclonal anti-GFAP → Cy3-labed anti-mouse IgG → polyclonal anti-MT-3 → Alexa488-labeled anti-rabbit IgG. The filter settings were as listed above for FITC/Alexa488 and Cy3.

### Statistical Analysis

Differences in mean Fe, Cu, and Zn concentrations in all six-color zones were analyzed by standard *t* test, assuming equal variance (Table 2). The following method was used to determine the abnormal admixture of copper and iron in the DN of FRDA: The Fe map of each case or control was displayed on a computer screen and segmented into six zones as described above. Regions of maximum Fe XRF were outlined on a superimposed clear plastic sheet that was placed over the computer screen, and Fe signals were collected from 25 randomly selected points. While the plastic sheet remained in place, Fe maps were exchanged by Cu maps, and Cu signals were collected from the same field. Concentrations of Cu and Fe were determined as described above. For each case or control, a mean Cu/Fe ratio was calculated from the 25 data points. Mean Cu/Fe ratios of the 10 FRDA patients and 13 controls were analyzed for statistically significant differences by standard *t* test. A significance level of *α* = 0.05 was used for all analyses and comparisons.

**Table 2:** Iron, copper, and zinc levels in the DN of 10 FRDA patients and 13 normal controls

Specimens	Zones					
	White	Red	Orange	Green	Light blue	Dark Blue
Iron						
FRDA	344 ± 159	276 ± 134	219 ± 101	160 ± 75	86 ± 35	42 ± 18
Normal	364 ± 117	297 ± 108	242 ± 85	178 ± 55	102 ± 33	55 ± 15
Copper						
FRDA	33 ± 18	24 ± 13	18 ± 10	14 ± 8	13 ± 5	12 ± 4
Normal	33 ± 13	24 ± 10	18 ± 9	14 ± 7	7 ± 5	–
Zinc						
FRDA	33 ± 19	26 ± 16	21 ± 13	16 ± 10	13 ± 5	5 ± 1
Normal	32 ± 16	23 ± 8	18 ± 7	13 ± 6	–	–

Results are expressed as micrograms per milliliter PEG/DMSO (mean ± standard deviation). The computer program rendered maps based on interquartile range. Metal maps were segmented into six zones of different color to allow a more detailed assessment of concentration gradients. White represents maximum XRF. Red, orange, green, light blue, and dark blue represent zones of progressively lower XRF intensity. In some specimens, the computer program did not resolve light and dark blue zones due to insufficient copper or zinc XRF

## Results

**Fig. 2:**
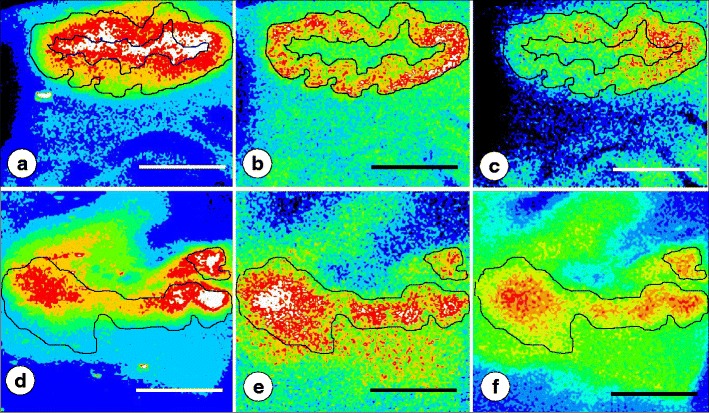
XRF maps of the DN in a normal control and a case of FRDA. **a**–**c** normal; **d**–**f** FRDA. The outlines of the Cu maps with peak signals (**b**, **e**) were transferred to the matching Fe and Zn maps. In the normal control (**a**–**c**), zones of maximum Cu and Zn are clearly demarcated from the peak Fe map (*white*). In FRDA (case FRDA5 F in Table 1), the normal ribbon-like Cu map has been replaced by several coalescing fields (**e**) that show extensive overlap with the peak Fe map (**d**). The distribution of Zn remains similar to that of Cu. *Bars*, 5 mm

Figure 1 shows the encapsulated metalloporphyrin standards in PEG/DMSO blocks, the matching Fe, Cu, and Zn XRF maps, and examples of regression analysis of XRF in counts/5 s as a function of metal concentrations. Figure 2 illustrates Fe, Cu, and Zn maps of the DN in a normal control and a case of FRDA. Despite the obvious collapse of the DN in FRDA, the signals of Fe, Cu, and Zn persist. In the control specimen, Fe XRF is at a maximum in the center of the DN (mean in micrograms per milliliter ± S.D., 346 ± 117) and declines in a gradient toward the gray matter ribbon and the surrounding white matter (Fig. 2a). In the same tissue block, the strongest Cu and Zn signals arise from a relatively narrow ribbon that corresponds to the gray matter of the nucleus (Fig. 2b, c). In the normal DN, regions of maximum Fe are demarcated from the Cu and Zn regions. In contrast, the DN in FRDA displays a widening of the Cu and Zn zones and extensive overlap with the region of maximum Fe XRF (Fig. 2d–f). In fields of maximal Fe XRF (white), the mean Cu/Fe ratio and standard deviation in normal controls (0.046 ± 0.012) are statistically significant (*p* = 0.04) from FRDA (0.058 ± 0.016). This data reflect a significant increase of Cu from 4.6 to 5.8 % of Fe in the same zone.

Table 2 shows the quantification of Fe, Cu, and Zn in six-colored zones along a concentration gradient within the DN. Mean concentrations in FRDA do not differ significantly from the normal state.

**Fig. 3:**
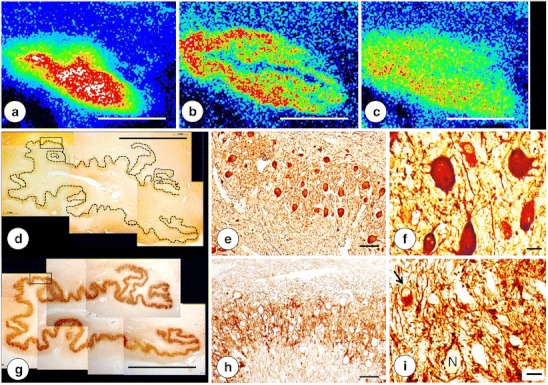
Fe, Cu, and Zn XRF maps of a normal DN and matching sections after recovery from PEG/DMSO. **a** Fe XRF; **b** Cu XRF; **c** Zn XRF. **d**–**f** Immunohistochemistry of NSE; **g**–**i** Immunohistochemistry of GAD. For clarity, the low-power composite of the DN gray matter ribbon in **d** was outlined by an *interrupted line*. The microphotographs in **e** and **h** correspond to the regions indicated by the *rectangles* in **d** and **g**, respectively. The XRF maps confirm the differential localization of Fe, Cu, and Zn. The DN gray matter ribbon, as shown by NSE (**d**) and GAD reaction products (**g**), matches the distribution of maximum Cu (**b**) and Zn (**c**), whereas Fe XRF places the bulk of Fe into the central white matter of the DN (**a**). Both immunohistochemical stains show the normal thickness of DN gray matter (250–300 μm). NSE reaction product visualizes large and small neurons of the normal DN (**e**–**f**). GAD reaction product labels axosomatic and axodendritic terminals, yielding negative images of DN nerve cells (**h**–**i**). The *arrow* in **i** points to a small neuron with GAD immunoreactivity in its cytoplasm. *N*, negative image of a neuron. *Bars*, **a**–**d** and **g** 5 mm; **e**, **h** 100 μm; **f**, **i** 20 μm

**Fig. 4:**
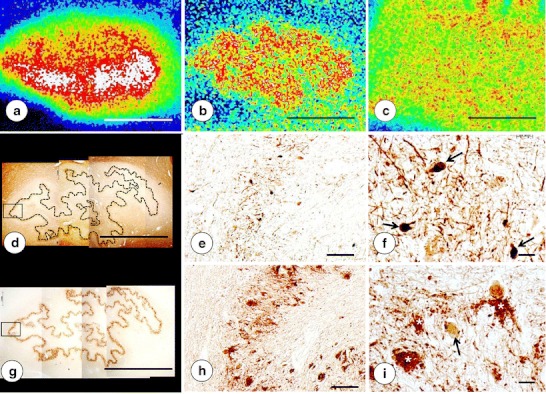
Fe, Cu, and Zn XRF maps of a DN in FRDA and matching sections after recovery from PEG/DMSO. **a** Fe XRF; **b** Cu XRF; **c** Zn XRF. **d**–**f** Immunohistochemistry of NSE; **g**–**i** Immunohistochemistry of GAD. For clarity, the low-power composite of the DN gray matter ribbon in **d** was outlined by an *interrupted line*. The microphotographs in **e** and **h** correspond to the regions indicated by the *rectangles* in **d** and **g**, respectively. Regions of maximal XRF for Fe (**a**), Cu (**b**), and Zn (**c**) show extensive overlap. Higher magnification confirms thinning of the DN to 100–120 μm and loss of large neurons (**e**, **h**). Small neurons are present in normal abundance (*arrows* in **f**). Atrophy of the DN gray matter is also evident following GAD immunohistochemistry (**g**–**i**). Negative images of large neurons are absent, but grumose degeneration retains GAD immunoreactivity (*white asterisks* in **i**). The *arrow* in **i** indicates a small intact GABA-ergic neuron with cytoplasmic GAD reaction product. Images derived from patient FRDA3, F in Table 1. *Bars*: **a**–**d**, **g** 5 mm; **e**, **h** 100 μm; **f**, **i** 20 μm

Figure 3 presents the alignment of Fe, Cu, and Zn maps with stained sections after transfer of the PEG/DMSO-infiltrated tissue blocks into paraffin. The Cu map (Fig. 3b) matches the major turns of the DN gray matter ribbon as revealed by NSE (Fig. 3d) and GAD immunohistochemistry (Fig. 3g). At higher magnifications of a defined region, the immunohistochemical stains reveal the normal neuropil of the DN (Fig. 3e, f, h, and i), including a small GABA-ergic neuron (Fig. 3i, arrow). A similarly processed specimen from a patient with FRDA is illustrated in Fig. 4. While still recognizable on the immunohistochemical stains of NSE and GAD, the greatly thinned gray matter ribbon of the DN now coincides with a much more diffuse Cu and Zn distribution (Fig. 4b, c). The stains for NSE (Fig. 4d–f) and GAD (Fig. 4g–i) of the outlined area of the DN gray matter reveal loss of large nerve cells (Fig. 4e, f) and grumose degeneration of the DN (Fig. 4h, i). NSE (Fig. 4f) and GAD (Fig. 4i) reaction products confirm the preservation of small neurons in the DN. Cytoplasmic GAD reactivity of one small nerve cell reveals its GABA-ergic nature (Fig. 4i, arrow).

**Fig. 5:**
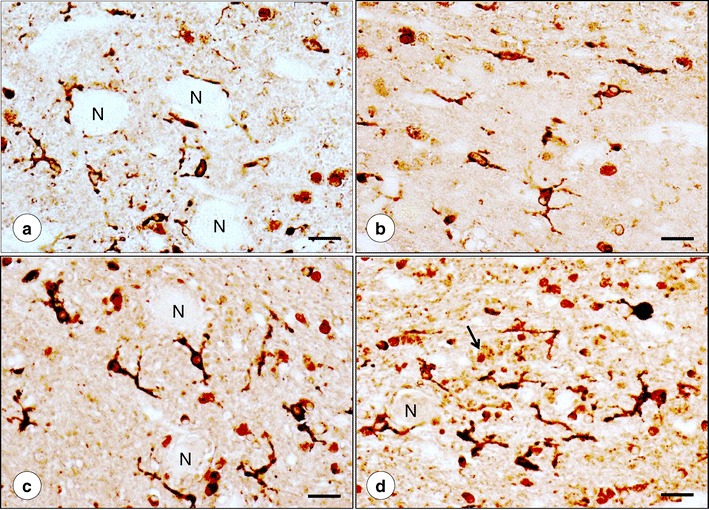
Ferritin immunohistochemistry of DN gray matter. **a**, **b** Normal control; **c**, **d** FRDA (case FRDA1, M in Table 1). In the normal DN gray matter, reaction product labels cells with the morphology of microglia (**a**, **b**) though the juxtaneuronal ferritin-positive cells in **a** may represent oligodendroglia [4]. At the junction to the hilar white matter (**b**), ferritin-positive cells are all microglia. In FRDA, ferritin-reactive microglia are larger (**c**) and more frequent about grumose degeneration (**d**). The *arrow* in **d** points toward grumose degeneration that also contains ferritin reaction product. The parent neuron of the dendrites surrounded by grumose degeneration is located to the left. *N*, negative images of neuronal cell bodies; *bars*, 20 μm

Figure 5 illustrates ferritin in the gray matter of the normal DN (Fig. 5a, b) and the DN of FRDA (Fig. 5c, d). Reaction product occurs mostly in microglia though some of the juxtaneuronal cells may be oligodendroglia (Fig. 5a). Microglia in FRDA are somewhat larger and plumper than normal (Fig. 5c), and grumose degeneration in FRDA (Fig. 5d) appears to generate a more prominent microglial response. The clusters constituting grumose degeneration also display ferritin reaction product.

**Fig. 6:**
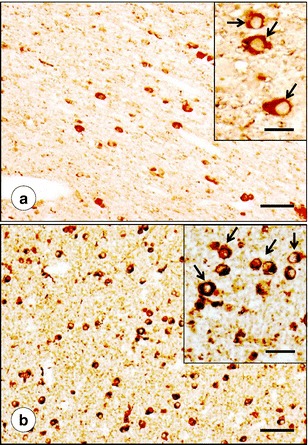
Ferritin immunohistochemistry of hilar white matter of the DN. **a** Normal control; **b** FRDA (case FRDA1, M in Table 1). In the normal hilar white matter, ferritin-positive cells are consistent with oligodendroglia (*arrows* in **a**, *inset*). Ferritin reaction product also labels oligodendroglia in the DN white matter of FRDA (**b**), but their density per unit area is higher, and their size is smaller (*arrows* in **b**, *inset*). *Bars*: **a**, **b** 50 μm; (**a** and **b** *insets*), 20 μm

Ferritin reaction product is present in the oligodendroglia of the DN hilus as shown in Fig. 6. In the normal state, these cells are larger than in FRDA and more widely spaced (Fig. 6a and inset). This separation is most likely due to the presence of normal myelinated fibers. In contrast, ferritin-reactive oligodendroglia in the DN hilus of FRDA are smaller and more tightly spaced. The increased density reflects loss of efferent axons and their myelin sheaths (Fig. 6b and inset).

**Fig. 7:**
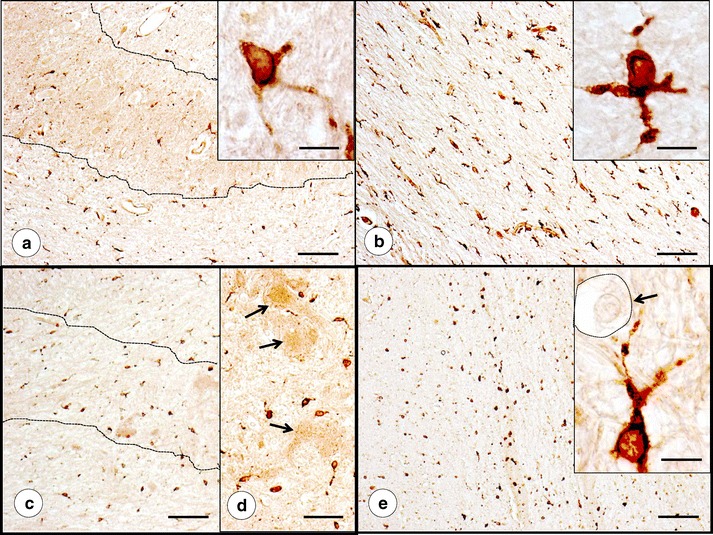
SOD immunohistochemistry of DN gray and white matter. **a**, **b**, Normal control; **c**–**e**, FRDA (case FRDA8, M in Table 1); **a**, **c**–**d** Gray matter (DN outlined by *interrupted lines*); **b**, **e** White matter. The plump SOD-reactive cells with sparse processes are consistent with microglia (*insets* in **a**, **b**, and **e**). These SOD-reactive cells are generally more frequent in the hilar white matter of the DN (**b**, **e**) than in the DN gray matter (**a**, **c**). Abundance, size, and shape of SOD-reactive cells in FRDA (**c**–**e**) do not differ from normal (**a**, **b**). SOD-reactive cells cluster around grumose degeneration in FRDA (*arrows* in **d**). The *inset* in **e** (*arrow and interrupted line*) shows a large SOD-negative oligodendrocyte in the immediate vicinity of a strongly SOD-reactive microglial cell. *Bars*: **a**–**c** and **e** 100 μm; *insets* in **a**, **b**, and **e** 10 μm (oil immersion optics); **d** 50 μm

Figure 7 displays positive-contrast immunohistochemistry of SOD in DN gray and white matter. SOD-reactive cells are similar in shape and abundance when control (Fig. 7a, b) and FRDA are compared (Fig. 7c, e). They are more frequent in the hilar white matter than in the gray matter of the DN. The SOD-reactive cells resemble oligodendroglia (Fig. 7b, e, insets). Figure 7e (inset, arrow) shows a negative image of a typical white matter oligodendrocyte adjacent to an SOD-positive cell. SOD-immunoreactive cells may be slightly more abundant around zones of grumose degeneration in FRDA (Fig. 7d).

**Fig. 8:**
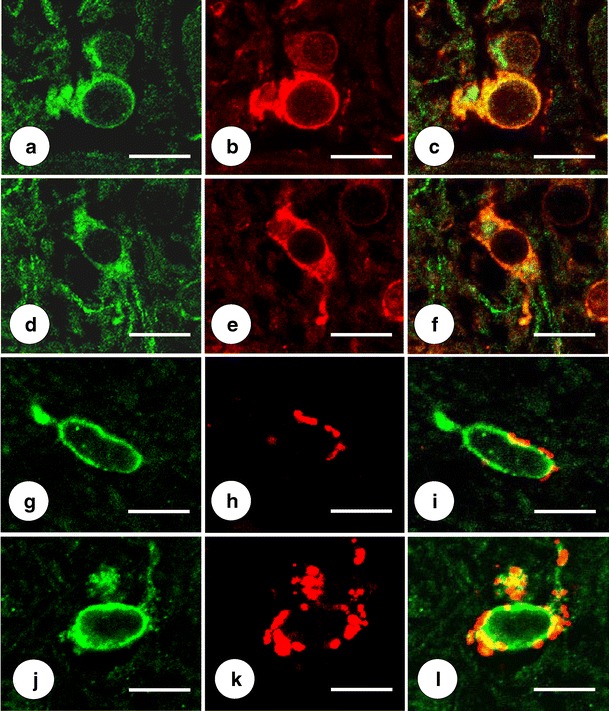
Double-label immunofluorescence of the pairs SOD/ferritin and SOD/CD68 in the DN of normal controls and two cases of FRDA. **a**–**c**, **g**–**i** Normal controls; **d**–**f** FRDA (case FRDA8, M in Table 1); **j**–**l** FRDA (case FRDA2, M in Table 1); **a**, **d** SOD (FITC, *green*); **b**, **e** Ferritin (Cy3, *red*); **c**, **f** Merged images. **g**, **j** SOD (Alexa488); **h**, **k** CD68 (Cy3); **i**, **l** merged images. SOD fluorescence in perikarya and proximal processes co-localizes with cytosolic ferritin (**c**, **f**). The SOD-immunoreactive cells also contain granular CD68 reaction product (**i**, **l**). In FRDA (**k**–**l**), CD68 reaction product is more abundant, suggesting microglial hypertrophy (see also Fig. 5c). Confocal microscopy at an optical slice thickness of 1 μm. *Bars*, 10 μm

Figure 8 confirms co-localization of SOD with cytosolic ferritin and CD68 in DN gray matter, supporting the identification of SOD-positive cells as microglia. All SOD-positive microglia contain ferritin and CD68 reaction products though not all ferritin- and CD68-reactive cells contain SOD. The abundant CD68 reaction product in the cell illustrated in Fig. 8k suggests microglial hypertrophy in FRDA that is also apparent by positive-contrast immunohistochemistry of ferritin (Fig. 5c). The strongly reactive oligodendroglia of the DN white matter (Fig. 6) are SOD-negative (not illustrated). In a separate double-label immunofluorescence procedure with anti-SOD and anti-GFAP, SOD-reactive cells were GFAP-negative, militating against the presence of the enzyme in astrocytes (not illustrated).

**Fig. 9:**
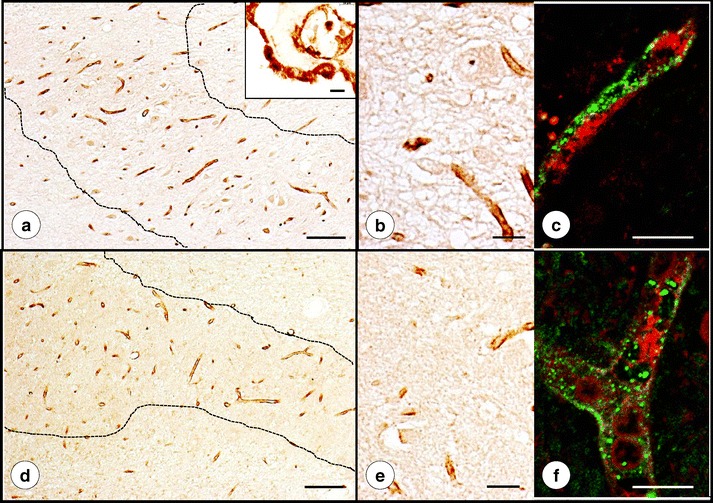
ATP7A immunohistochemistry and immunofluorescence of the DN in a normal control and FRDA. **a**–**c** Normal control; **d**–**f** FRDA (case FRDA2, M in Table 1). In **a** and **d**, the approximate boundaries of the DN gray matter are outlined by *interrupted lines*. Positive-contrast reaction product labels capillary walls that are much more abundant in the DN neuropil than in the white matter of hilus or fleece, reflecting the generally higher vascularity of gray matter. FRDA (**d**–**f**) does not differ from the control (**a**–**c**). The *inset* in **a** shows reaction product in epithelial cells of the choroid plexus on the same slide, serving as an internal positive control. Higher power resolution (**b** and **e**) shows the granular nature of ATP7A reaction product in vessel walls, which is more apparent on double-label fluorescence of ATP7A (FITC, *green*) and RCA-1 (TRITC, *red*) (**c** and **f**). Fluorescent reaction product of ATP7A is present in granules or vesicles with a diameter of less than 0.5 μm and does not occur uniformly in all areas of the vessel wall. Confocal microscopy at an optical slice thickness of 1 μm. *Bars*: **a**, **d** 100 μm; **b**, **e**, and *inset* in **a** 20 μm; **c**, **f** 10 μm

Figure 9 shows ATP7A protein by positive-contrast immunohistochemistry and in combined immunofluorescence with RCA-1 lectin affinity fluorescence. In DN gray and white matter, ATP7A reaction product is prominent in vessel walls. Strong immunoreactivity of choroid plexus epithelium in the same section serves as a positive control (Fig. 9a, inset). Higher vascularity of gray matter explains the greater number of reactive vessels in the DN ribbon than in the adjacent white matter, but there are no obvious differences between control and FRDA. Confocal fluorescence microscopy of sections doubly stained for ATP7A and carbohydrate chains (by TRITC-labeled RCA-1) confirms the localization of the copper-carrying protein in vessel walls. Reaction product of ATP7A occurs in small discontinuous packets with a diameter of less than 0.5 μm (Fig. 9c, f).

**Fig. 10:**
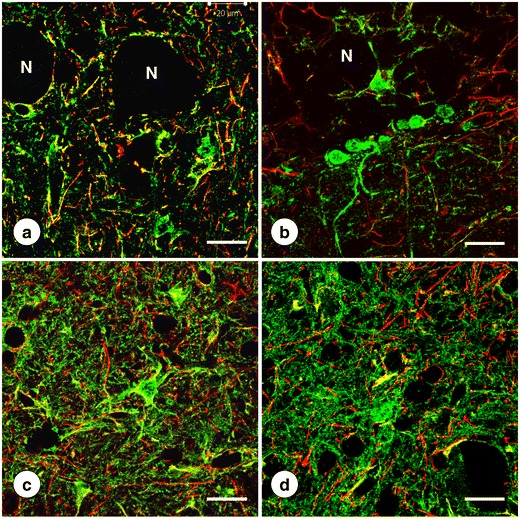
Double-label immunofluorescence of MT-1/2, MT-3, and GFAP in the DN. **a**, **b** Normal control; **c**, **d** FRDA (FRDA8, M in Table 1); **a**, **c** MT-1/2; **b**, **d** MT-3. The MTs are shown by green fluorescence, GFAP by red fluorescence. Neuronal loss in FRDA (**c**) and (**d**) is apparent by the absence of nerve cell voids (*N*). MT-1/2 and MT-3-containing astrocytic cell bodies show no GFAP reaction product. Only a few glial processes display co-localization of MT-1/2 and GFAP (**a**, **c**) or MT-3 and GFAP (**b**, **d**). In FRDA, both MT-1/2 and MT-3 show a greater abundance of finely granular green reaction product. Confocal microscopy at an optical slice thickness of 1 μm. *Bars*, 20 μm

Figure 10 illustrates the changes in MT-1/2 and MT-3 in the DN due to FRDA. The green finely granular MT reaction products are consistent with localization in astrocytes and their processes. The MT-1/2- and MT-3-immunoreactive cytoplasm of astrocytic cell bodies is GFAP-negative, whereas processes display some co-localization. In FRDA, fibrous gliosis is evident by red GFAP fluorescence (Fig. 10c, d). MT-1/2 and MT-3 fluorescence contributes to DN gliosis in FRDA by an abundance of delicate processes, but the disease does not cause a greater degree of co-expression of GFAP and the metallothioneins in comparison with the normal state.

## Discussion

### Application of XRF to Metal Measurements in Tissue Volumes

The XRF unit was originally developed for surface analysis [16–18], but the introduction of homogeneous metal standards (Fig. 1) now allows measurements based on volume. PEG has unusual properties when mixed with aqueous solutions due to its amphipathic nature [19]. Inorganic salts of Fe, Cu, and Zn are unsuitable for the development of metal standards, but Fe-III-, Cu-II-, and Zn-II-mesoporphyrins dissolve readily in liquefied PEG/DMSO. Progressive infiltration of fixed tissue samples by PEG/DMSO at 60°C displaces all tissue water, and XRF signals can be used to quantify Fe, Cu, and Zn in terms of micrograms per milliliter. Due to high water content of brain tissue, the results of this study approximate previous data on these metals in tissue samples that were expressed as micrograms per gram wet or dry weight [6–9].

Metal maps and quantification by XRF provide a major advantage over assays of tissue extracts. The method shows metal concentrations in situ and in context with the microscopic anatomy of the tissue. Metals in the DN are not homogeneously distributed and display nonidentical concentration gradients of Fe, Cu, and Zn (Fig. 2). Rapid processing after fixation is especially important for the study of Zn because this metal is subject to rapid redistribution due to autolysis [20]. To some degree, this artifact is also true for Cu. Prolonged storage of cerebellar *slices* ultimately causes the disappearance of Cu and Zn from XRF maps, and three FRDA cases were rejected from further analysis. Persistence of Fe fluorescence even after extended storage in fixative is likely due to metal localization in the shell of holoferritin. In successfully processed control specimens (Fig. 2), Cu and Zn appear in comparable locations (Fig. 2b, c), presumably because these metals are normally present in neurons and terminals.

### Persistence of Fe, Cu, and Zn in the DN of FRDA; and Increasing Admixture of Fe and Cu

Friedreich's ataxia does not increase or decrease the net amounts of Fe, Cu, or Zn (Table 2). Koeppen et al. [4] assayed total Fe in digests of dissected DN and found values in FRDA that were in the normal range. Western blots, however, revealed a larger contribution of light (L) ferritin subunits to holoferritin, suggesting accelerated ferritin biosynthesis due to persistent excess of free Fe. Localization and persistence of Fe have clinical implications. Signal hypointensity on T2-weighted magnetic resonance images (MRI) in FRDA [21, 22] detects bulk Fe in DN white matter, and it is unlikely that the more diffuse distribution of Cu and Zn in FRDA has measurable effects on MRI signals. Routine MRI of the DN in FRDA at high magnetic field strengths is only now emerging, and none of the patients listed in Table 1 had targeted visualization of this gray matter structure. The DN pathology reported here represents the morphological end stage of FRDA. Death of an FRDA patient from cardiomyopathy before onset of ataxia is uncommon. In such cases, the DN may still be entirely normal [2].

The two outstanding abnormalities in the DN of FRDA are the persistence of Fe, Cu, and Zn in and about the DN and the increasing admixture of Cu, Zn, and Fe signals (Figs. 2 and 4). It is evident that destruction of DN neurons and synaptic terminals releases Cu and Zn, which then diffuse into the neuropil and enter the adjacent Fe-rich white matter. The clinical importance of adding Cu and Zn to the Fe-rich regions of the DN in FRDA is unknown. Though Cu ions may equal Fe ions in the ability to generate oxygen radicals [23, 24], the immunohistochemical observations provide no insight into potential oxidative damage induced by Cu or Zn. Without an efficient local control mechanism, the endogenous Cu concentrations in the DN (12–33 μg/ml; Table 2) would be sufficient to cause tissue damage. The previously reported intracerebral injection of Cu sulfate at 0.5 μg/0.1 ml [12], equaling 19.9 μg/ml of Cu, causes necrosis [12], but this result is not directly comparable to endogenous Cu that is largely bound to proteins. Inorganic Cu [11, 12] is known to be more toxic than Cu bound to proteins [13]. It is likely that the main form in the DN of FRDA is protein-bound Cu.

### Correlation of XRF Maps and Histology

Atrophy of the DN in FRDA, as shown by immunohistochemistry of NSE (Fig. 4d–f) and GAD (Fig. 4g–i), must be correlated with Cu and Zn redistribution rather than with Fe only. Despite its main localization in hilar white matter of the DN, Fe in the gray matter ribbon is still substantially higher than the maximum Cu and Zn concentrations in the same location (Table 2). Therefore, Fe in the DN gray matter cannot be ignored, especially in light of frataxin loss in the DN of patients with FRDA [1]. Ferritin is a surrogate marker of disturbed Fe metabolism, but immunohistochemistry with anti-ferritin shows only modest changes in abundance and size of microglia in DN gray matter (Fig. 5c, d). Grumose degeneration appears to attract ferritin-positive microglia (Fig. 5d), and it is of interest that the abnormal clusters of GABA-ergic terminals also contain ferroportin [4]. It is evident that oligodendroglia in the DN hilus express ferritin constitutively, and crowding of small ferritin-containing cells in the central white matter of the DN may be a secondary event without relevance to the pathogenesis of FRDA (Fig. 6b).

### Control Mechanisms of Fe, Cu, and Zn in FRDA

Findings illustrated in Figs. 5, 7, 8, and 10 suggest that the potentially toxic admixture of Fe and Cu in FRDA generates a defensive response in microglia and astrocytes. The universal reaction to surges in free Fe in mammalian tissues is the translational stimulation of ferritin biosynthesis through the interaction of iron-responsive elements in the 5′-untranslated region of messenger ribonucleic acid and iron-regulatory proteins 1 and 2. Sequestration of ferric iron inside the shell of holoferritin is an efficient mechanism by which Fe-catalyzed generation of oxygen radicals is mitigated. The immediate defense against free or loosely bound brain Fe occurs in microglia. Juxtaneuronal cells in the DN (Fig. 5a) and oligodendroglia in the DN white matter (Fig. 6) may involve a ferritin receptor [25], but the response of these cells to the FRDA disease process appears limited in comparison with gray matter microglia (Fig. 5c, d).

The potential endogenous excess of Cu and Zn in FRDA is limited to the DN and occurs over many years during the course of the patient's illness. While Cu and Zn dysmetabolism can probably not be measured by attention to a single Cu- or Zn-responsive protein in analogy to ferritin, SOD, ATP7A, and the metallothionein isoproteins are promising candidates [26]. More is known about brain Cu control than about the fate of cerebral Zn (20). Brain Cu turnover in adult rats is extremely slow [27], and retention of the metal in the DN of patients with FRDA is not surprising. The mechanism of this Cu conservation is not known. Insight into cerebral Cu homeostasis is largely based on experimental Cu *deficiency* rather than excess [28, 29]. Extracerebral organs respond to Cu deficiency by reduced SOD levels that reflect a posttranscriptional mechanism. Brain SOD activity and protein levels in adult rats do not decline despite a significant reduction in Cu concentration [28]. Cu incorporation into SOD relies on a copper chaperone for SOD (CCS). It is expressed in brain and increases in response to lowered brain Cu levels [30]. Peculiarly, the lack of CCS in genetically modified mice causes severely lowered levels of brain Cu, SOD protein, and SOD activity [30]. Therefore, it may be assumed that Cu homeostasis in the normal human DN also involves CCS–SOD interaction [31], but a role of these proteins in the Cu redistribution in the DN of FRDA patients remains to be established.

Immunohistochemistry and immunofluorescence of SOD and ATP7A (Figs. 7, 8, and 9) provide only inconclusive evidence that the FRDA-damaged DN undergoes changes due to Cu excess. Clustering of SOD-positive cells about regions of grumose degeneration (Fig. 7d) suggests a response to Cu release from corticonuclear terminals. The presence of SOD reaction product in microglia, as shown in Figs. 7 and 8, is at variance with the neuronal localization in the human CNS reported by others [32]. Positive-contrast and immunofluorescent reaction products suggest that Cu bound to SOD and Fe sequestered in holoferritin share the same localization (Fig. 8). It may be reasonable to propose that SOD activity and Fe sequestration in holoferritin provide an efficient antioxidant mechanism in microglia. Granular ATP7A fluorescence in vessel walls (Fig. 9c, f) is consistent with vesicular trafficking between the trans-Golgi network and the plasma membrane [33], including endothelial cells [34, 35]. ATP7A primarily serves Cu uptake [34, 35] rather than the return of the metal to the bloodstream. Figure 9 suggests that the mechanism of Cu uptake in the DN of FRDA remains unchanged.

Metallothionein immunohistochemistry has been used to detect a biological response to Cu excess in the brain [36], though the experiments were unusual. Sheep were first Cu-fed and then treated over a period of time with the Cu-chelator ammonium tetrathiomolybdate. The Cu-chelator complex entered the brain and raised total Cu in selected regions three- to four-fold. The steepest increase occurred in cerebellum (from 16.7 to 60.8 μg/g). While this Cu excess caused no neuronal damage, it strongly stimulated expression of metallothioneins in astroglia, as shown by immunohistochemistry with an antibody to MT1 and MT2. It is generally accepted that astrocytes strongly express MT-1/2 and MT-3 [37]. Blaauwgeers et al. [38] reported that the cellular localizations of MT and GFAP in normal human brain are mutually exclusive, implying MT expression in a subset of astrocytes. The observations shown here (Fig. 10) are consistent with this interpretation. It may also be important that DN gliosis in FRDA did not change the relative abundance of GFAP and MT-1/2 or MT-3 proteins (Fig.10). MT occurs in at least four isoforms that respond to several metals other than Cu and Zn and to various nonmetal stimuli. The observations illustrated in Fig. 10c, d do not prove that an excess of Cu, Zn, or both cause the greater abundance of MT-positive fibers in FRDA. Upregulation of brain MT occurs in disorders that are not obviously related to metals [37], and caution is necessary in the interpretation of increased MT reaction product in the DN of FRDA. Nevertheless, the participation of MT-1/2 and MT-3 in the glial response to FRDA that is independent of GFAP expression indicates an intact biochemical and cellular machinery for Cu and Zn detoxification. It cannot be assumed, however, that Cu and Zn remain innocuous through the course of the patient's illness. Protein analysis by Western blot may be a more informative method to detect changes in SOD, ATP7A, CCS, and the metallothioneins.

### Fe and Cu in Other Diseases of the DN

Redistribution of Fe and Cu in the DN nucleus is not unique to FRDA. Among the hereditary ataxias, spinocerebellar ataxia type 3 is of immediate interest because neuronal loss, survival of small neurons, and grumose degeneration of the DN and integrity of the inferior olivary nuclei are similar to FRDA. In two suitable cases, XRF showed a similar collapse of the DN and merging of Fe, Cu, and Zn signals. Grumose degeneration, loss of large neurons, and preservation of small nerve cells also occur in the DN of progressive supranuclear palsy (PSP). XRF mapping of five specimens of PSP showed no Cu or Zn signals due to prolonged storage in fixative. The distribution of Fe, however, displayed collapse of the DN and retention of maximum Fe XRF in the hilus of the nucleus that strongly resembled FRDA.

### A Rationale for Therapy of FRDA Based on Insights into Metal Dysmetabolism

The "intimate relationship" of Fe and Cu metabolism [39] is likely as true for the central nervous system as it is for other organs and offers new research avenues in FRDA and for therapeutic approaches. Beyond a direct damaging effect of free Cu on the tissues of the DN, it is also possible that metal impacts the fate of Fe in the same location. Measuring Fe- and Cu-mediated oxidative damage to the neurons of the DN in human autopsy tissues remains a challenge. There is little doubt that atrophy of large DN neurons is primary [2] but the precise mechanism of their selective destruction remains elusive. Chelation therapy to remove Fe [21] and possibly Cu [40] may protect these cells against downstream damage. Study of the surviving small neurons [2] may offer additional insight into the pathogenesis of the DN lesion in FRDA. Small nerve cells in the DN are heterogeneous and not exclusively GABA-ergic. In rodents, some of the smaller neurons of the cerebellar nuclei are glycinergic [41] or use other transmitters in their connections. FRDA does not affect the GABA-ergic dentato-olivary tract in humans [2], but the fate of small non-GABA-ergic neurons is unknown. Small neurons in the DN may have an intrinsic resistance to frataxin deficiency or tolerance to local Cu and Zn excess.

A possible therapy to mitigate Cu toxicity to the DN is the systemic administration of pregabalin [42]. Marmolino and Manto [42] delivered small amounts of free Cu into the nucleus interpositus of rats by reverse microdialysis. The animals showed increased excitability of the contralateral motor cortex that could be inhibited by pretreatment with pregabalin. The experiments addressed the functional consequences of local Cu excess and differed from previous studies that only sought to determine the necrotizing effects of Cu on brain tissue [11, 12]. Delivery of small amounts of Cu into cerebellar nuclei is not directly comparable to the slow redistribution of Cu in the human DN during FRDA, but the results still provide a reasonable rationale for a clinical trial with pregabalin.
